# Chlorogenic and Caftaric Acids in Liver Toxicity and Oxidative Stress Induced by Methamphetamine

**DOI:** 10.1155/2014/583494

**Published:** 2014-07-20

**Authors:** Khaled M. M. Koriem, Rowan E. Soliman

**Affiliations:** ^1^Medical Physiology Department, Medical Research Division, National Research Centre, El-Buhouth Street, Dokki, Giza 12622, Egypt; ^2^Advanced Medical and Dental Institute (AMDI), Universiti Sains Malaysia (USM), No. 6 Tingkat 1, Persiaran Seksyen 4/9, Bandar Putra Bertam, 13200 Kepala Batas, Pulau Pinang, Malaysia; ^3^Faculty of Pharmacy, Cairo University, Kasr El-Aini Street, P.O. Box 11562, Cairo 11787, Egypt

## Abstract

Methamphetamine intoxication can cause acute hepatic failure. Chlorogenic and caftaric acids are the major dietary polyphenols present in various foods. The aim of this study was to evaluate the protective role of chlorogenic and caftaric acids in liver toxicity and oxidative stress induced by methamphetamine in rats. Thirty-two male albino rats were divided into 4 equal groups. Group 1, which was control group, was injected (i.p) with saline (1 mL/kg) twice a day over seven-day period. Groups 2, 3, and 4 were injected (i.p) with methamphetamine (10 mg/kg) twice a day over seven-day period, where groups 3 and 4 were injected (i.p) with 60 mg/kg chlorogenic acid and 40 mg/kg caftaric acid, respectively, one day before methamphetamine injections. Methamphetamine increased serum aspartate aminotransferase, alanine aminotransferase, alkaline phosphatase, bilirubin, cholesterol, low-density lipoprotein, and triglycerides. Also, malondialdehyde in serum, liver, and brain and plasma and liver nitric oxide levels were increased while methamphetamine induced a significant decrease in serum total protein, albumin, globulin, albumin/globulin ratio, brain serotonin, norepinephrine and dopamine, blood and liver superoxide dismutase, and glutathione peroxidase levels. Chlorogenic and caftaric acids prior to methamphetamine injections restored all the above parameters to normal values. In conclusion, chlorogenic and caftaric acids before methamphetamine injections prevented liver toxicity and oxidative stress where chlorogenic acid was more effective.

## 1. Introduction

Methamphetamine (METH) is a potent addictive psychostimulant, commonly referred to as “speed,” “crystal,” “crank,” “go,” and “ice.” It is reportedly being abused by approximately 35 million people worldwide [1]. It has been reported that approximately 5.8% of Americans, aged 12 years or older, have used METH at least once in their lifetime [2]. A dramatic increase in METH-related emergency department visits is alarming, with >50% involving young adults aged 18–34 years [3]. The relative ease of METH's availability, coupled with its toxicity, has resulted in increased numbers of associated medical complications and fatalities [3, 4]. Chronic use and acute METH intoxication can cause substantial medical consequences, including kidney (rhabdomyolysis, myoglobinuria, and acute renal failure [5, 6]), liver (acute hepatic failure [7, 8] and centrilobular liver damage [9]), lungs (pulmonary edema and shortness of breath [10]), cardiovascular (tachycardia, atrioventricular arrhythmias, myocardial ischemia, and hypertension [11, 12]), cerebrovascular (hemorrhages, strokes, and seizures [13, 14]), and psychiatric problems (psychosis, persecutory delusions, persistent visual and auditory hallucinations, depression, anxiety, aggressiveness, social isolation, psychomotor dysfunction [15, 16], and suicidal ideation [17, 18]). Individuals with METH-related disorders have a higher risk of schizophrenia than those with other drug use disorders and these effects explained by shared etiological mechanisms are involved in the development of schizophrenia [19]. The METH showed increased risk of Parkinson's disease compared to that of both the matched appendicitis and the matched cocaine groups [20]. The toxic effects of METH on the brain are well-known and are linked to oxidative stress; little information is available about the oxidative stress induced by METH in other organs. Tokunaga et al. [21] studied changes in renal function and found that repeated METH administration induced oxidative DNA injury to the kidney, as a chronic or subacute influence.

METH causes a massive release of dopamine in the brain by degenerating dopaminergic terminals and damaging dopaminergic neurons. Dopamine then reacts with molecular oxygen to form reactive oxygen species (ROS) where ROS and free radicals have attracted increasing attention over the past decade. ROS are continuously produced during normal physiologic events and they can easily initiate the peroxidation of membrane lipids, leading to the accumulation of lipid peroxides. However, they are removed by antioxidant defense mechanisms. There is a balance between the generation of ROS and inactivation of ROS by the antioxidant system in organisms. Under pathological conditions, ROS are overproduced and result in oxidative stress. Imbalance between ROS and antioxidant defense mechanisms leads to oxidative modification in cellular membrane or intracellular molecules [22, 23].

New treatment challenges have arisen with the increased use of METH [24] in the last decade. However, there are currently no pharmacological treatments for the wide range of symptoms associated with METH-related problems, possibly because of the lack of understanding of METH-induced toxicity. The medicinal plants provide a new, available, and cheap source for developing new drugs nowadays. Natural products account for more than 40% of all pharmaceuticals on the market today, where from 1941 to 2002, over 50% of all the drugs, or new drug entities, available for cancer treatment were derived from natural resources [25]. Chlorogenic and caftaric acids are the esters forms of caffeic acid. These compounds are the major dietary polyphenols present in various foods and beverages. The similarities in the metabolic patterns observed for caffeic, chlorogenic, and caftaric acids suggest that esterification does not influence the metabolism of caffeic acid by the gut microbiota [26]. Chlorogenic and caftaric acids were demonstrated to be more powerful antioxidants in a number of different systems [27, 28]. Chlorogenic and caftaric acids are good substrates of polyphenol oxidases, and under certain conditions they may undergo oxidation in plant tissues or products of plant origin [29, 30]. Chlorogenic acid is one of the major phenolic compounds identified in peach and in prunes while caftaric acid is found in grape. Chlorogenic acid has antihypertensive effect [31]; it also has a protective effect in neuroinflammatory condition on dopaminergic neurons [32]. On the other hand, caftaric acid has a hyaluronidase inhibitory activity, antioxidant property, and enhancement of insulin secretion [33, 34].

The purpose of the present study was to discover a natural adjunctive new therapeutic agent to treat liver toxicity and oxidative stress induced by METH. To accomplish this, we measured several liver function and oxidative stress parameters to determine if METH induces oxidative stress in liver tissue and if this stress could be prevented by the use of chlorogenic and caftaric acids.

## 2. Materials and Methods

### 2.1. Materials

Methamphetamine was obtained from Novartis Pharmaceutical Company, Egypt. All other chemicals, chlorogenic and caftaric acids, were obtained from Sigma-Aldrich GmbH, Sternheim, Germany. All Kit reagents were obtained from Biomerieux Company, France, through local supplier.

#### 2.1.1. Animals

Male albino (Sprague Dawley) rats weighing 130–140 g were obtained from animal house colony of National Research Centre, Egypt. They were kept under the hygienic conditions and well balanced diet and water. The experiments were carried out according to the National Regulations on Animal Welfare and Institutional Animal Ethical Committee (IAEC).

#### 2.1.2. Experimental Design

A total number of thirty-two SD rats were chosen for such a study and the quantity of samples meets the basic requirement of statistics. The animals were housed in a controlled temperature (~22°C) and humidity (~55%) animal facility, with a 12-hour light and dark cycle. The animals had unlimited access to rodent chow and water and were utilized after 1 day of acclimatization. All animal procedures were conducted under an animal protocol approved by the Institutional Animal Care and Use Committee of National Research Centre, Egypt.

The rats were divided into four equal groups as follows. Group 1 which was the control group was injected (i.p) with saline (1 mL/kg) twice a day over seven-day period. Groups 2, 3, and 4 were injected (i.p) with METH (10 mg/kg) twice a day over seven-day period, where group 3 was injected once (i.p) with 60 mg/kg chlorogenic acid [35] and group 4 was injected once (i.p) with 40 mg/kg caftaric acid [36], respectively, one day before METH injections.

All rats were anesthetized 24 hours after the last METH injection by inhalation with diethyl ether solution. All rats were massed at the beginning and the end of the study.

#### 2.1.3. Blood Sampling and Handling

Blood samples were collected from retroorbital plexus of rats using capillary tubes into clean centrifuge tubes. Part of blood sample was collected in the presence of using EDTA as an anticoagulant for blood while the other part of the blood sample was allowed to coagulate and centrifuged at 4000 rpm for 15 min to separate blood serum which was stored at −20°C.

#### 2.1.4. Tissue Preparation

The animals were decapitated and then dissected, whereby the liver and brain tissues were obtained, washed in cold saline, and dried between filter papers. They were weighed, homogenized, and kept at −80°C for further investigation; 0.5 gm of liver and brain tissues was dissolved in 2.5 mL of Tris buffer solution and then homogenated in the homogenizer at a speed of 2500 rpm for exactly 30 min using ice bath. Then they were centrifuged (−4°C) for exactly 20 min at 7000 rpm, which separated the supernatants, which were used for antioxidant activities determination.

### 2.2. Methods

#### 2.2.1. Liver Function

Serum aspartate aminotransferase (AST) and alanine aminotransferase (ALT) were determined according to the method of Reitman and Frankel [37]. Serum total bilirubin determination was performed with the Walters and Gerarde [38] method. Serum alkaline phosphatase (ALP) was determined by the colorimetric method of Kind and King [39]. The determination of serum total protein was performed according to the method of Gornall et al. [40]. Determination of serum albumin (Alb) was according to the method of Drupt [41]. Serum globulin (Glob) and (Alb)/Glob ratio were estimated [42].

#### 2.2.2. Lipids Fractions

Total cholesterol was determined using the enzymatic method according to Allain et al. [43]. Determination of serum low-density lipoproteins (LDL) was adopted and described by Steinberg [44]. Serum triglycerides were determined according to the method of Fossati and Prencipe [45]. Serum high-density lipoprotein (HDL) was determined according to the method described by Fruchart et al. [46].

#### 2.2.3. Antioxidants Enzymes

Determination of superoxide dismutase (SOD) levels in blood, liver, and brain was estimated based on the method of Suttle [47]. Blood and liver glutathione peroxidase (GPx) levels were estimated using the method of Paglia and Valentine [48]. A colorimetric assay was used for detecting lipid peroxidation (MDA) levels in serum, liver, and brain tissues according to the method of Esterbauer et al. [49]. Plasmanitrate and nitrite concentrations as an indicator of nitric oxide generation were analyzed according to the method of Moshage et al. [50].

#### 2.2.4. Brain Cerebral Cortex Neurotransmitters

Estimation of serotonin (5-hydroxytryptamine; 5-HT), norepinephrine (NE), and dopamine (DA) in brain cerebral cortex was carried out according to the method described by Ciarlone and Smudski [51].

#### 2.2.5. Histopathological and Histochemical Examinations

Specimens of liver were fixed in 10% neutral formalin solution and then processed for routine embedding in paraffin. Blocks were sectioned at a thickness of 5 *μ*m and stained with hematoxylin and eosin for histopathological examination.

Other liver sections were stained with periodic acid-Schiff (PAS) for the histochemical examination which was performed under light microscopy and documented by an Olympus microphotocamera.

### 2.3. Statistical Analysis

The results were expressed as mean ± standard error (SE). Statistical significance was determined through one-way analysis of variance (ANOVA), followed by Student's *t*-test. *P* values less than 0.05 were considered statistically significant. *P ≤ 0.05 represents significant difference compared to control (−ve control) and **P ≤ 0.01 represents highly significant differences compared to control (−ve control). ^a^ *P* ≤ 0.05 represents significant difference compared to METH (+ve control) group. ^b^ *P* ≤ 0.01 represents highly significant difference compared to METH (+ve control) group.

## 3. Results

In the present study, there were no observable differences of body weight, food, or drink intake during experimental period of the study.

### 3.1. Liver Function

The results presented in Table 1 indicated that METH caused a significant increase (*P* < 0.05) in serums AST, ALT, ALP, and bilirubin levels in rats. The pretreatment with chlorogenic or caftaric acid prior to METH injections inhibits (*P* > 0.05) the increase in AST, ALT, ALP, and bilirubin as compared to the METH-injected rats where chlorogenic acid was more potent than caftaric acid.

Table 2 revealed the protective role of chlorogenic or caftaric acid on serum total protein, albumin, globulin levels, and albumin/globulin ratio in METH-injected rats. It is obvious that METH caused a significant decrease (*P* < 0.05) in serum total protein, albumin, globulin levels, and albumin/globulin ratio as compared with control rats. While either chlorogenic or caftaric acid pretreated before METH injections induced a significant increase (*P* > 0.05) in total protein albumin, albumin, globulin levels, and albumin/globulin ratio in METH-injected rats as compared with that in METH-injected rats, on the other hand, chlorogenic or caftaric acid pretreated METH-injected rats revealed insignificant increase (*P* > 0.05) in serum albumin and albumin/globulin ratio while there was a significant increase in serum total protein (*P* < 0.05) compared to METH-injected rats group. METH-injected rats + chlorogenic or caftaric acid exhibited a highly significant increase (*P* < 0.01) in serum globulin where chlorogenic acid was more effective than caftaric acid.

### 3.2. Lipid Fractions

The data presented in Table 3 showed that METH induced a significant increase (*P* < 0.05) in serum cholesterol on the contrary;chlorogenic or caftaric acid pretreatment to METH-injected rats exhibited an insignificant increase (*P* > 0.05) in serum cholesterol compared to control rats. The level of serum low-density lipoprotein (LDL) in normal rats after METH injection then treatment with chlorogenic or caftaric acid was exhibited in Table 3. Analysis of the changes which occurred in LDL after METH injections showed a significant increase (*P* < 0.05) in serum LDL, but administration with chlorogenic or caftaric acid to rats injected with METH revealed an insignificant increase (*P* > 0.05) in LDL compared to control rats. Analysis of serum triglycerides in normal, METH-injected, or pretreated with either chlorogenic or caftaric acid toMETH-injected rats was found in Table 3. Serum triglycerides after METH injection revealed a significant increase (*P* < 0.05) in serum triglycerides while chlorogenic or caftaric acid pretreated to METH-injected rats revealed an insignificant increase (*P* > 0.05) in serum triglycerides compared to control rats where chlorogenic acid was more potent than caftaric acid.

### 3.3. Antioxidants Enzymes

It was found that a significant decrease (*P* < 0.05) in blood and liver SOD and GPx were recorded in association with METH injections and the data is tabulated in Tables 4 and 5. On the contrary, the pretreatment with chlorogenic or caftaric acid to METH-injected rats induced insignificant increase (*P* > 0.05) in blood and liver SOD and GPx when compared with control rats. On the other hand, chlorogenic or caftaric acid to METH-injected group showed a significant increase (*P* < 0.05) in blood and liver SOD but a highly significant increase (*P* < 0.01) in blood and liver GPx compared to METH injected rats. METH caused a significant increase (*P* < 0.05) in serum and liver malondialdehyde (MDA) and plasma and liver nitric oxide (NO) as compared with the control rats. A significant decrease (*P* > 0.05) was observed in serum and liver MDA as well as plasma and liver NO in eithergroup pretreated with chlorogenic or caftaric acid before METH injections as compared with those injected with METH-injected rats (Tables 4 and 5) where chlorogenic acid was more potent than caftaric acid.

### 3.4. Brain Cerebral Cortex Neurotransmitters

Table 6 revealed the effect of chlorogenic and caftaric acidson serotonin, norepinephrine, dopamine, and MDA in the brain cerebral cortex of METH-injected rats. It is clear that METH induced a highly significant decrease (*P* < 0.01) in brain neurotransmitters (serotonin, norepinephrine, and dopamine) while increased significantly brain MDA. The pretreatment of both chlorogenic and caftaric acids to METH-injected rats restores (*P* > 0.05) the above parameters to approach the normal values.

### 3.5. Histology Results

**Figure 1:**
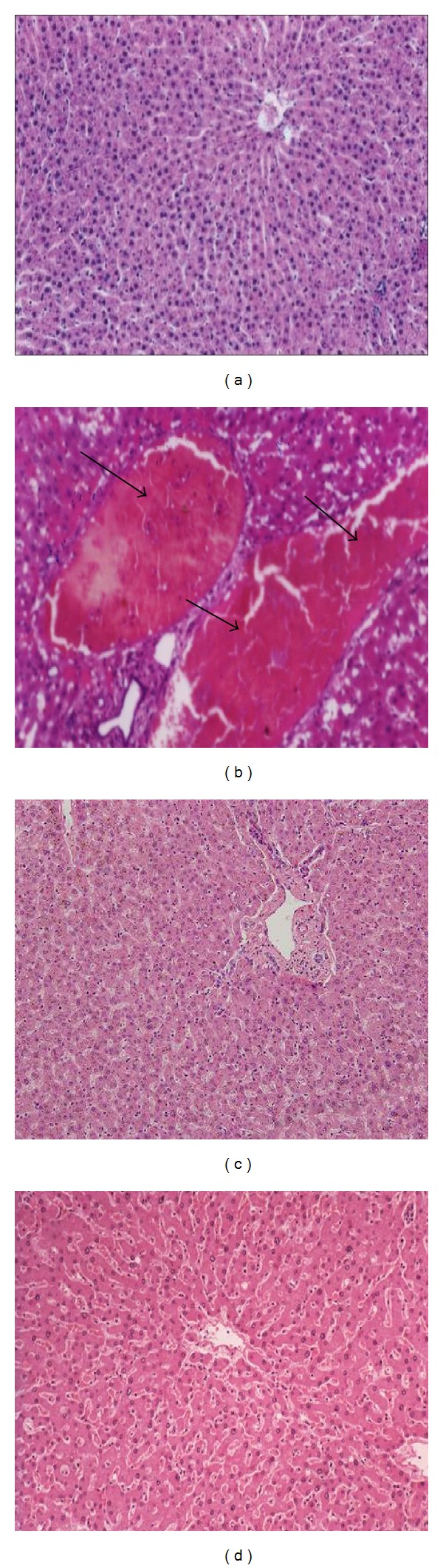
(a) The control group with preserved hepatic architecture (H&E ×200). (b) METH injections caused by a hoop of oedema in the periportal area (arrowhead), which compressed the surrounding hepatocytes. The intracytoplasm vacuolation was found (H&E ×400). (c) Pretreatment of chlorogenic acidwith preserved hepatic lobular architecture. The hepatocytes are within normal limit and preserved its plate pattern. Liver almost returns to the normal pattern (H&E ×200). (d) Pretreatment of caftaric acid to METH injected rats with large reserved hepatic lobular architecture and the liver almost returns to the normal pattern (H&E ×200).

Figure 1 revealed the histopathology results in METH injected rats as well as chlorogenic or caftaric acid potentially pretreated groups. The structure of the control liver showed normal hepatocytes, vascular sinusoids, and centrilobular vein (Figure 1(a)). METH injections caused a hoop of oedema in the periportal area, which compressed the surrounding hepatocytes (Figure 1(b)). Examination of liver sections of rats pretreated with chlorogenic or caftaric acid prior to METH injections showed preserved hepatic lobular architecture. The hepatocytes were within normal limit and preserved its plate pattern. Liver almost returned to the normal pattern (Figures 1(c) and 1(d)).

### 3.6. Histochemical Results

**Figure 2:**
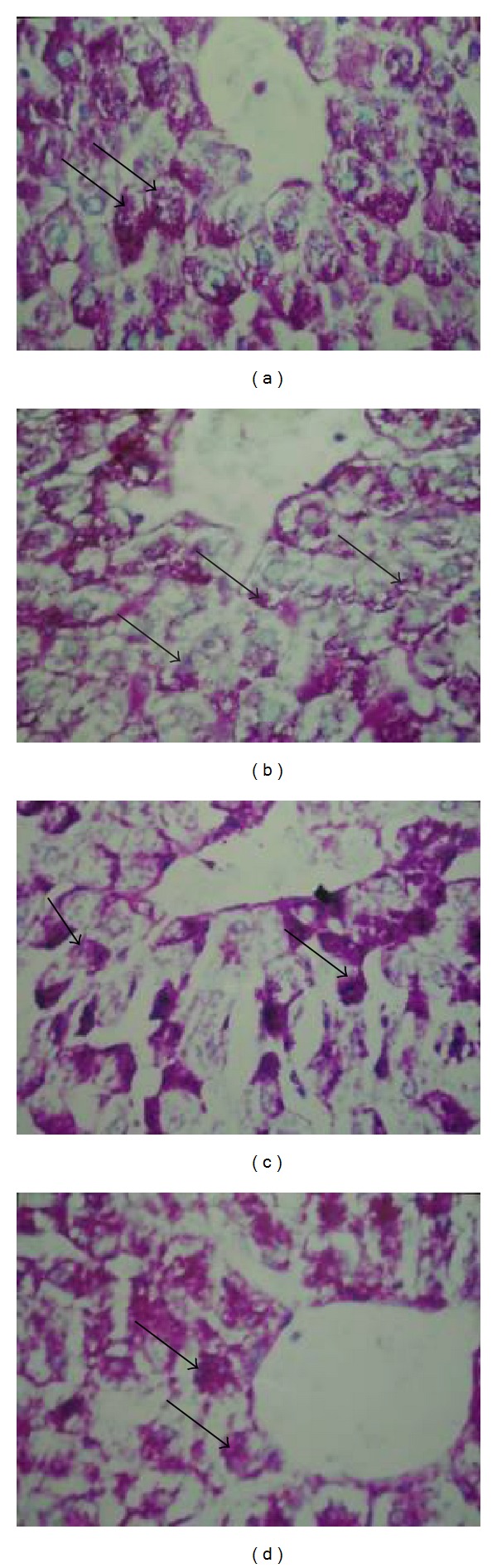
A photomicrograph of section of liver showing the following. (a) Control: the glycogen particles appear accumulated (arrowhead) in the cytoplasm. (b) Rats injected with METH showing the polysaccharides inclusions that displayed diffuse stain ability (arrowhead). A few number of the hepatocytes display dense stain ability compared to the others. (c) Rats injected with METH in combination with chlorogenic acid showing the polysaccharides inclusions (arrowhead) that appear more or less as control (PAS ×300). (d) Rats injected with METH in combination with caftaric acid showing the polysaccharides inclusions (arrowhead) that appear more or less as control (PAS ×300).

Examination of liver of control rats stained with Periodic Acid Schiff's (PAS) technique showed the abundance of polysaccharide materials in the hepatocytes. The polysaccharides particles appear accumulated at one side of the cytoplasm, leaving the other side. In the liver lobule, the hepatocytes at the periphery appear markedly rich in glycogen particles if compared with the pericentral cells (Figure 2(a)). Successive daily METH injections induced diffuse stain ability of the positive PAS materials of the hepatocytes of the METH-treated rats. A few of the hepatocytes displayed denser stain ability than the others (Figure 2(b)). In rats' pretreated with chlorogenic or caftaric acid before METH-injections, the positive PAS materials of the hepatocytes appeared more or less as normal (Figures 2(c) and 2(d)).

## 4. Discussion

Methamphetamine (METH) toxicity is quite prevalent worldwide due to its euphoric effects, wide availability, and relatively low cost. The multiple adverse effects of METH involve perturbation in dopamine, serotonin, glutamate [52], nitric oxide [53], and noradrenaline [54]. Initially, METH causes a massive release of dopamine in the brain by inhibiting monoamine oxidase activity and dopamine uptake [55]. With higher doses, however, it causes dopamine depletion by degenerating dopaminergic terminals, damaging dopaminergic neurons, and decreasing dopamine transporter numbers [56]. Dopamine then reacts with molecular oxygen to form reactive oxygen species (ROS), such as hydrogen peroxide, superoxide, and hydroxyl free radicals resulting in a condition known as oxidative stress [57]. Oxidative stress is believed to play a crucial role in METH-induced toxicity in the brain and other tissues, as evidenced by findings in previous studies. However, a comparison of oxidative effects of METH in different organs has not been sufficiently studied previously [21, 58].

METH increased significantly creatinine and creatinine phosphokinase (CPK), while serum minerals, potassium, calcium, and phosphorus levels decreased, so METH induced renal dysfunction with renal tubule damage; this damage is related to leakage of CPK from the skeletal muscle as an index of skeletal muscle damage following METH injections, also oxidative DNA damage was induced by repeated administration of METH [21]. Moreover, total antioxidant levels were lower while MDA levels were higher in the METH-treated group at postnatal day 21 in both males and females offspring when pregnant female Wistar rats were given METH (5 mg/kg bwt/day; sc) from gestation days (GD) 8 to 22 [55].

The mechanism of protection of chlorogenic acid or caftaric acid was dependant on the antioxidant activity of these acids to reduce the oxidative stress produced by METH injections. The aim of the present study was to evaluate a protective role of chlorogenic acid or caftaric acid in liver toxicity and oxidative stress which occurred in METH injections in male albino mice.

The clinical and diagnostic values associated with the changes in blood enzyme concentrations such as AST, ALT, ALP, and serum bilirubin have long been recognized [59, 60]. Increased levels of these diagnostic markers of hepatic function in METH injections in rats are implicative of the degree of hepatocellular dysfunction caused by METH injections. Comparatively lower levels of these parameters in the pretreatment withchlorogenic or caftaric acidgroup to METH-injected group show the ability of these acids to protect the liver against harmful effects of METH injections.

Albumin is the most abundant circulatory protein and its synthesis is a typical function of normal liver cells. Low levels of albumin have been reported in the serum of patients and animals with hepatocellular cancer [61]. The fall in the serum albumin levels could probably contribute to the low total protein levels observed in METH injected rats. Considerably higher albumin and total protein levels were seen in chlorogenic or caftaric acid pretreated groups as compared to METH group, indicating that one of the mechanisms by which chlorogenic or caftaric acid exhibit their protective effect during cancer is by enhancing the levels of albumin and thereby total protein levels. Thus, based on our preliminary biochemical findings, we suggest possible preventive effects of chlorogenic or caftaric acid into rats' hepatotoxicity shown in METH injections.

METH injections increased significantly serum cholesterol, LDL, and triglycerides, while chlorogenic or caftaric acid pretreatment directed serum cholesterol, LDL, and triglycerides to the normal levels. This effect may be related to chlorogenic or caftaric acid which protects low-density lipoproteins from oxidation. Lipids targeted for cellular metabolism are mobilized from the intestine as follows: (1) triglycerides-rich chylomicrons; (2) triglycerides-rich very-low-density lipoproteins, which are subsequently converted to cholesterol-rich LDL; and (3) cholesterol-phospholipid-rich HDL, which removes cholesterol from peripheral cells and transports it to the liver [62]. Such findings were in agreement with that of de Sotillo and Hadley [63] who found that chlorogenic acid decreased plasma cholesterol and triacylglycerols concentrations by 44% and 58%, respectively, as it did in liver triacylglycerols concentrations (24%). Also, Wan et al. [64] mentioned that chlorogenic acid has hypocholesterolemic effect which leads to other secondary beneficial effects such as atheroscleroprotective, cardioprotective, and hepatoprotective functions. Moreover, Harnafi et al. [65] reported that caftaric acid reduced the atherogenic index and LDL/HDL-C ratio by 88% and 94%, respectively. Caftaric acid reduced also liver total cholesterol and triglycerides by 50% and 58%, respectively.

METH injections decreased significantly blood and liver GPx and SOD activities but increased serum, liver, and brain MDA, as well as plasma and liver NO. On the contrary, chlorogenic or caftaric acidpretreated to METH-injected rats increased blood and liver GPx and SOD activities but decreased serum and liver MDA and plasma and liver NO. This observation could be related to the antioxidant effect of chlorogenic or caftaric acid. Such findings were accepted with that of Koriem et al. [66] who found that caffeic acid increased glutathione and glutathione peroxidase levels while it decreased malondialdehyde and catalase levels in brain, liver, and kidney tissues exposed to METH. Also, Sato et al. [67] reported in vitro and in vivo antioxidant prosperities of chlorogenic and caffeic acids. Furthermore, Cejudo-Bastante et al. [68] reported antioxidant effect of caftaric acid. Moreover, Huang et al. [69] stated that METH abusers have persistently higher systemic oxidative stress throughout early abstinence. The compromised SOD as well as elevated CAT activity and GSH levels may act together as a compensatory mechanism to counteract excessive oxidative stress induced by METH. In addition, Melo et al. [70] reported that lipid peroxidation and NO were significantly higher in the retina and blood plasma of the METH-treated rats while the total antioxidant levels (SOD, GPx, and catalase) were significantly lower in both retina and blood plasma of the METH-treated rats. Also, Ajjimaporn et al. [71] showed that METH enhances lipid peroxidation and decreases the antioxidant-reduced glutathione (GSH) together with an inhibition of mitochondrial complex-I activity. Pretreatment with zinc markedly prevents the increase of lipid peroxidation and provides mitochondrial protection by scavenging free radicals and increasing mitochondrial GSH. Finally, Solhi et al. [72] proved that prolonged use of methamphetamine exerts oxidative stress on the body and enhances lipid peroxidation.

In the current study, METH induced a highly significant decrease in brain neurotransmitters (serotonin, norepinephrine, and dopamine), where METH induces dopamine depletion by degenerating dopaminergic terminals, damaging dopaminergic neurons, and decreasing dopamine transporter numbers [56]. The pretreatment of both chlorogenic and caftaric acids to METH-injected rats restores the neurotransmitters to approach the normal values. These effects are related to cytoprotective effect of chlorogenic and caftaric acids against brain catecholaminergic cells toxicity [66, 73].

Histopathological and histochemical studies of liver tissues of normal, METH-injected group, and chlorogenic or caftaric acid+ METH-injected group indicate that either acid has cytoprotective property.

## 5. Conclusion

Data from the present study highlights METH fatal and toxic effects on liver to induce liver toxicity and oxidative stress in male albino mice. The pretreatment of chlorogenic acid or caftaric acidhas shown a significant improvement in liver toxicity and oxidative stress in METH-injected rats evidenced in histological and histochemical examinations. However, further clinical studies are warranted to establish its effectiveness and it will be interesting to see whether chlorogenic acid or caftaric acidreverses existing METH induced liver toxicity and oxidative stress, like in real case scenarios.

## Figures and Tables

**Table 1:** Effect of chlorogenic and caftaric acids on serums AST, ALT, total bilirubin, and ALP of METH-injected rats.

Groups	Parameters			
	AST (U/L)	ALT (U/L)	Total bilirubin (mg/dL)	ALP (U/L)
Control	123.1 ± 3.85	62.5 ± 2.48	0.56 ± 0.08	210.5 ± 6.81
METH group	140.2 ± 0.72∗	82.0 ± 1.96∗∗	0.75 ± 0.05∗∗	241.2 ± 7.15∗∗
Chlorogenic acid + METH group	125.4 ± 4.17^a^	60.1 ± 2.9^b^	0.58 ± 0.04^b^	215.1 ± 6.59^b^
Caftaric acid + METH group	128.3 ± 3.94^a^	65.0 ± 2.87^b^	0.60 ± 0.07^a^	223.0 ± 4.86^a^

Data presented as mean ± SE. Number of animals = 8 per group.

∗Significant change (*P* ≤ 0.05) compared to control rats.

∗∗Highly significant change (*P* ≤ 0.01) compared to control rats.

^a^Significant change (*P* ≤ 0.05) compared to METH group.

^b^Highly significant change (*P* ≤ 0.01) compared to METH group.

**Table 2:** Effect of chlorogenic and caftaric acids on serum total protein, albumin, globulin, and albumin/globulin ratio of METH-injected rats.

Groups	Parameters			
	Total protein (g/dL)	Albumin (g/dL)	Globulin (g/dL)	Alb/glob. ratio
Control	7.64 ± 0.72	3.76 ± 0.45	3.88 ± 0.25	0.97 ± 0.05
METH group	6.10 ± 0.35∗	2.81 ± 0.17∗	3.29 ± 0.17∗	0.85 ± 0.03∗
Chlorogenic acid + METH group	8.16 ± 0.93^a^	3.96 ± 0.48^a^	4.18 ± 0.18^b^	0.95 ± 0.04^a^
Caftaric acid + METH group	7.75 ± 0.68^a^	3.86 ± 0.65^a^	3.89 ± 0.25^a^	0.90 ± 0.07

Data presented as mean ± SE. Number of animals = 8 per group.

∗Significant change (*P* ≤ 0.05) compared to control rats.

∗∗Highly significant change (*P* ≤ 0.01) compared to control rats.

^a^Significant change (*P* ≤ 0.05) compared to METH group.

^b^Highly significant change (*P* ≤ 0.01) compared to METH group.

**Table 3:** Effect of chlorogenic and caftaric acids on lipid fractions of METH-injected rats.

Groups	Parameters			
	Cholesterol (mg/dL)	LDL (mg/dL)	Triglycerides (mg/dL)	HDL (mg/dL)
Control	98.2 ± 3.46	40.7 ± 1.95	47.1 ± 2.01	48.1 ± 1.87
METH group	107.5 ± 2.86∗	46.1 ± 1.86∗	52.4 ± 1.93∗	50.9 ± 2.03
Chlorogenic acid + METH group	99.5 ± 3.89^a^	41.8 ± 1.75^a^	48.2 ± 1.68^a^	47.6 ± 1.68
Caftaric acid + METH group	101.4 ± 4.05	43.5 ± 2.04	50.3 ± 2.14	47.8 ± 1.92

Data presented as mean ± SE. Number of animals = 8 per group.

∗Significant change (*P* ≤ 0.05) compared to control rats.

^a^Significant change (*P* ≤ 0.05) compared to METH group.

**Table 4:** Effect of chlorogenic and caftaric acids on blood SOD and GPx, serum MDA, and plasma NO of METH-injected rats.

Groups	Parameters			
	SOD (U/mL)	GPx (U/L)	MDA (*μ*mol/L)	NO (*μ*mol/L)
Control	260 ± 8.39	6250 ± 82.95	3.51 ± 0.26	35.8 ± 1.78
METH group	236.9 ± 7.86∗	5985 ± 98.71∗	5.76 ± 0.54∗∗	50.1 ± 2.25∗∗
Chlorogenic acid + METH group	258.7 ± 8.63^a^	6240 ± 86.93^a^	3.54 ± 0.38^b^	37.0 ± 1.98^b^
Caftaric acid + METH group	265.1 ± 7.91^a^	6435 ± 100.14^b^	3.71 ± 0.42^b^	40.5 ± 1.86^a^

Data presented as mean ± SE. Number of animals = 8 per group.

∗Significant change (*P* ≤ 0.05) compared to control rats.

∗∗Highly significant change (*P* ≤ 0.01) compared to control rats.

^a^Significant change (*P* ≤ 0.05) compared to METH group.

^b^Highly significant change (*P* ≤ 0.01) compared to METH group.

**Table 5:** Effect of chlorogenic and caftaric acids on liver SOD, GPx, MDA, and NO of METH-injected rats.

Groups	Parameters			
	SOD (mU/mg tissue)	GPx (mU/mg tissue)	MDA (nmol/100 mg tissue)	NO (*µ*mol/mg tissue)
Control	137.85 ± 12.19	3.69 ± 0.16	29.57 ± 3.16	4.83 ± 1.51
METH group	65.87 ± 9.42∗∗	1.12 ± 0.41∗∗	56.85 ± 4.15∗∗	8.24 ± 1.29∗∗
Chlorogenic acid + METH group	136.87 ± 12.64^b^	3.58 ± 0.20^b^	30.65 ± 2.47^b^	4.95 ± 1.32^b^
Caftaric acid + METH group	129.56 ± 13.16^a^	3.48 ± 0.37^b^	34.25 ± 3.85^a^	5.21 ± 1.49^a^

Data presented as mean ± SE. Number of animals = 8 per group.

∗Significant change (*P* ≤ 0.05) compared to control rats.

∗∗Highly significant change (*P* ≤ 0.01) compared to control rats.

^a^Significant change (*P* ≤ 0.05) compared to METH group.

^b^Highly significant change (*P* ≤ 0.01) compared to METH group.

**Table 6:** Effect of chlorogenic and caftaric acids on serotonin, norepinephrine, dopamine, and MDA in the brain cerebral cortex of METH-injected rats.

Groups	Parameters			
	Serotonin (*µ*g/gm tissue)	Norepinephrine (*µ*g/gm tissue)	Dopamine (*µ*g/gm tissue)	MDA (nmol/mg tissue)
Control	0.56 ± 0.05	2.49 ± 0.19	0.27 ± 0.01	34.85 ± 5.16
METH group	0.32 ± 0.10∗∗	1.02 ± 0.13∗∗	0.14 ± 0.03∗∗	47.13 ± 8.63∗
Chlorogenic acid + METH group	0.54 ± 0.08^b^	2.46 ± 0.08^b^	0.25 ± 0.05^b^	36.25 ± 6.09^a^
Caftaric acid + METH group	0.50 ± 0.05^a^	2.44 ± 0.11^b^	0.21 ± 0.04^a^	38.54 ± 5.42^a^

Data presented as mean ± SE. Number of animals = 8 per group.

∗Significant change (*P* ≤ 0.05) compared to control rats.

∗∗Highly significant change (*P* ≤ 0.01) compared to control rats.

^a^Significant change (*P* ≤ 0.05) compared to METH group.

^b^Highly significant change (*P* ≤ 0.01) compared to METH group.

## References

[1] Office of Applied Studies (2004). Primary methamphetamine/amphetamine treatment admissions: 1992–2002. *The DASIS Report*.

[2] NSDUH Report (2007). *Methamphetamine Use*.

[3] Office of Applied Studies (2003). Emergency Department Trends from Drug Abuse Warning Network, Final Estimates 1995-2002. *DHHS Publication*.

[4] Gonzales R, Mooney L, Rawson RA (2010). The methamphetamine problem in the United States. *Annual Review of Public Health*.

[5] Richards JR, Johnson EB, Stark RW, Derlet RW (1999). Methamphetamine abuse and rhabdomyolysis in the ED: a 5-year study. *The American Journal of Emergency Medicine*.

[6] Shima N, Miyawaki I, Bando K (2011). Influences of methamphetamine-induced acute intoxication on urinary and plasma metabolic profiles in the rat. *Toxicology*.

[7] Jones AL, Simpson KJ (1999). Review article: mechanisms and management of hepatotoxicity in ecstasy (MDMA) and amphetamine intoxications. *Alimentary Pharmacology and Therapeutics*.

[8] Carvalho F, Remião F, Soares ME, Catarino R, Queiroz G, Bastos ML (1997). d-Amphetamine-induced hepatotoxicity: Possibie contribution of catecholamines and hyperthermia to the effect studied in isolated rat hepatocytes. *Archives of Toxicology*.

[9] Kamijo Y, Soma K, Nishida M, Namera A, Ohwada T (2002). Acute liver failure following intravenous methamphetamine. *Veterinary and Human Toxicology*.

[10] Mooney LJ, Glasner-Edwards S, Rawson RA, Ling W, Roll JM, Rawson RA, Ling W, Shoptaw S (2009). Medical effects of methamphetamine use. *Methamphetamine Addiction: From Basic Science to Treatment*.

[11] Yu Q, Larson DF, Watson RR (2003). Heart disease, methamphetamine and AIDS. *Life Sciences*.

[12] Albertson TE, Derlet RW, van Hoozen BE (1999). Methamphetamine and the expanding complications of amphetamines. *Western Journal of Medicine*.

[13] Darke S, Kaye S, McKetin R, Duflou J (2008). Major physical and psychological harms of methamphetamine use. *Drug and Alcohol Review*.

[14] Homer BD, Solomon TM, Moeller RW, Mascia A, DeRaleau L, Halkitis PN (2008). Methamphetamine abuse and impairment of social functioning: a review of the underlying neurophysiological causes and behavioral implications. *Psychological Bulletin*.

[15] Scott JC, Woods SP, Matt GE (2007). Neurocognitive effects of methamphetamine: a critical review and meta-analysis. *Neuropsychology Review*.

[16] Zweben JE, Cohen JB, Christian D (2004). Psychiatric symptoms in methamphetamine users. *The American Journal on Addictions*.

[17] Glasner-Edwards S, Marinelli-Casey P, Hillhouse M, Ang A, Mooney LJ, Rawson R (2009). Depression among methamphetamine users: association with outcomes from the methamphetamine treatment project at 3-year follow-up. *Journal of Nervous and Mental Disease*.

[18] Meredith CW, Jaffe C, Ang-Lee K, Saxon AJ (2005). Implications of chronic methamphetamine use: a literature review. *Harvard Review of Psychiatry*.

[19] Callaghan RC, Cunningham JK, Allebeck P (2012). Methamphetamine use and schizophrenia: a population-based cohort study in California. *The American Journal of Psychiatry*.

[20] Callaghan RC, Cunningham JK, Sykes J, Kish SJ (2012). Increased risk of Parkinson’s disease in individuals hospitalized with conditions related to the use of methamphetamine or other amphetamine-type drugs. *Drug and Alcohol Dependence*.

[21] Tokunaga I, Kubo S, Ishigami A, Gotohda T, Kitamura O (2006). Changes in renal function and oxidative damage in methamphetamine-treated rat. *Legal Medicine*.

[22] Duh P, Tu Y, Yen G (1999). Antioxidant activity of water extract of haring jury (*Chrysanthemum morifolium* Ramat). *LWT-Food Science and Technology*.

[23] Büyükokuroğlu ME, Gülçin I, Oktay M, Küfrevioğlu OI (2001). In vitro antioxidant properties of dantrolene sodium. *Pharmacological Research*.

[24] Shrem MT, Halkitis PN (2008). Methamphetamine abuse in the United States: contextual, psychological and sociological considerations. *Journal of Health Psychology*.

[25] Newman DJ, Cragg GM, Snader KM (2003). Natural products as sources of new drugs over the period 1981–2002. *Journal of Natural Products*.

[26] Gonthier MP, Remesy C, Scalbert A (2006). Microbial metabolism of caffeic acid and its esters chlorogenic and caftaric acids by human faecal microbiota in vitro. *Biomedicine and Pharmacotherapy*.

[27] Meyer AS, Donovan JL, Pearson DA, Waterhouse AL, Frankel EN (1998). Fruit hydroxycinnamic acids inhibit low density lipoprotein oxidation in vitro. *Journal of Agricultural and Food Chemistry*.

[28] Fukumoto LR, Mazza G (2000). Assessing antioxidant and prooxidant activities of phenolic compounds. *Journal of Agricultural and Food Chemistry*.

[29] Kerry N, Rice-Evans C (1998). Peroxynitrite oxidises catechols to o-quinones. *FEBS Letters*.

[30] Bassil D, Makris DP, Kefalas P (2005). Oxidation of caffeic acid in the presence of L-cysteine: isolation of 2-S-cysteinylcaffeic acid and evaluation of its antioxidant properties. *Food Research International*.

[31] Zhao Y, Wang J, Ballevre O, Luo H, Zhang W (2012). Antihypertensive effects and mechanisms of chlorogenic acids. *Hypertension Research*.

[32] Shen W, Qi R, Zhang J (2012). Chlorogenic acid inhibits LPS-induced microglial activation and improves survival of dopaminergic neurons. *Brain Research Bulletin*.

[33] Raso GM, Pacilio M, di Carlo G, Esposito E, Pinto L, Meli R (2002). In-vivo and in-vitro anti-inflammatory effect of Echinacea purpurea and Hypericum perforatum. *Journal of Pharmacy and Pharmacology*.

[34] Barrett B (2003). Medicinal properties of Echinacea: a critical review. *Phytomedicine*.

[35] Gao R, Lin Y, Liang G, Yu B, Gao Y (2014). Comparative pharmacokinetic study of chlorogenic acid after oral administration of lonicerae japonicae flos and shuang-huang-lian in normal and febrile rats. *Phytotherapy Research*.

[36] Arimoto-Kobayashi S, Zhang X, Yuhara Y, Kamiya T, Negishi T, Okamoto G (2013). Chemopreventive effects of the juice of *Vitis coignetiae* Pulliat on two-stage mouse skin carcinogenesis. *Nutrition and Cancer*.

[37] Reitman S, Frankel S (1957). A colorimetric method for the determination of serum glutamic oxalacetic and glutamic pyruvic transaminases. *The American Journal of Clinical Pathology*.

[38] Walters MI, Gerarde HW (1970). An ultramicromethod for the determination of conjugated and total bilirubin in serum or plasma. *Microchemical Journal*.

[39] Kind PR, King EJ (1954). Estimation of plasma phosphatase by determination of hydrolysed phenol with amino-antipyrine. *Journal of Clinical Pathology*.

[40] Gornall AG, Bardawill CJ, David MM (1949). Determination of serum proteins by means of the biuret reaction.. *The Journal of biological chemistry*.

[41] Drupt F (1974). Determination of serum albumin by bromocresol green. *Pharmaceutical Biology*.

[42] Latner AL (1975). Clinical biochemistry. *Cantarow and Trumper*.

[43] Allain CC, Poon LS, Chan CS, Richmond W, Fu PC (1974). Enzymatic determination of total serum cholesterol. *Clinical Chemistry*.

[44] Steinberg D (1981). Metabolism of lipoproteins at the cellular level in relation to atherogenesis. *Lipoproteins, Atherosclerosis and Coronary Heart Disease*.

[45] Fossati P, Prencipe L (1982). Serum triglycerides determined colorimetrically with an enzyme that produces hydrogen peroxide. *Clinical Chemistry*.

[46] Fruchart J-, Kora I, Cachera C, Clavey V, Duthilleul P, Moschetto Y (1982). Simultaneous measurement of plasma apolipoproteins A-I and B by electroimmunoassay. *Clinical Chemistry*.

[47] Suttle NF (1986). Copper deficiency in ruminants; recent developments. *Veterinary Record*.

[48] Paglia DE, Valentine WN (1967). Studies on the quantitative and qualitative characterization of erythrocyte glutathione peroxidase. *The Journal of Laboratory and Clinical Medicine*.

[49] Esterbauer H, Schaur RJ, Zollner H (1991). Chemistry and biochemistry of 4-hyroxynonenal, malonaldehyde and related aldehydes. *Free Radical Biology and Medicine*.

[50] Moshage H, Kok B, Huizenga JR, Jansen PLM (1995). Nitrite and nitrate determinations in plasma: a critical evaluation. *Clinical Chemistry*.

[51] Ciarlone AE, Smudski JW (1977). Lidocaine's influence on the accumulation and depletion rates of mouse brain amines. *Journal of Dental Research*.

[52] Burrows KB, Yamamoto BK, Herman BH (2003). Methamphetamine neurotoxicity: roles for glutamate, oxidative processes and metabolic stress. *Glutamate and Addiction*.

[53] Cadet JL, Herman BH (2003). Roles of glutamate, nitric oxide, oxidative stress, and apoptosis in the neurotoxicity of methamphetamine. *Glutamate and Addiction*.

[54] Fleckenstein AE, Gibb JW, Hanson GR (2000). Differential effects of stimulants on monoaminergic transporters: pharmacological consequences and implications for neurotoxicity. *European Journal of Pharmacology*.

[55] Melo P, Rodrigues LG, Pinazo-Durán MD, Tavares MA (2005). Methamphetamine and lipid peroxidation in the rat retina. *Birth Defects Research A*.

[56] Giros B, Jaber M, Jones SR, Wightman RM, Caron MG (1996). Hyperlocomotion and indifference to cocaine and amphetamine in mice lacking the dopamine transporter. *Nature*.

[57] Stumm G, Schlegel J, Schäfer T (1999). Amphetamines induce apoptosis and regulation of bcl-x splice variants in neocortical neurons. *The FASEB Journal*.

[58] Burrows KB, Yamamoto BK, Herman BH (2003). Methamphetamine neurotoxicity: roles for glutamate, oxidative processes and metabolic stress. *Glutamate and Addiction*.

[59] Martin P, Friedman LS, Friedman L, Keeffe E (1998). Management of chronic liver-disease-Preface. *Handbook of Liver Disease*.

[60] Plaa GL, Hewitt WR, Hayes WA (1989). Detection and evaluation of chemical induced liver injury. *Principles and Methods of Toxicology*.

[61] Gray GE, Meguid MM (1990). Can total parenteral nutrition reverse hypoalbuminemia in oncology patients?. *Nutrition*.

[62] Formica JV (1995). Review of the biology of quercetin and related bioflavonoids. *Food and Chemical Toxicology*.

[63] de Sotillo DVR, Hadley M (2002). Chlorogenic acid modifies plasma and liver concentrations of: cholesterol, triacylglycerol, and minerals in (*fa/fa*) Zucker rats. *The Journal of Nutritional Biochemistry*.

[64] Wan C, Wong CN, Pin W (2013). Chlorogenic acid exhibits cholesterol lowering and fatty liver attenuating properties by up-regulating the gene expression of PPAR-*α* in hypercholesterolemic rats induced with a high-cholesterol diet. *Phytotherapy Research*.

[65] Harnafi H, Ramchoun M, Tits M (2013). Phenolic acid-rich extract of sweet basil restores cholesterol and triglycerides metabolism in high fat diet-fed mice: a comparison with fenofibrate. *Biomedicine and Preventive Nutrition*.

[66] Koriem KMM, Abdelhamid AZ, Younes HF (2013). Caffeic acid protects tissue antioxidants and DNA content in methamphetamine induced tissue toxicity in Sprague Dawley rats. *Toxicology Mechanisms and Methods*.

[67] Sato Y, Itagaki S, Kurokawa T (2011). In vitro and in vivo antioxidant properties of chlorogenic acid and caffeic acid. *International Journal of Pharmaceutics*.

[68] Cejudo-Bastante MJ, Durán-Guerrero E, Natera-Marín R, Castro-Mejías R, García-Barroso C (2013). Characterisation of commercial aromatised vinegars: Phenolic compounds, volatile composition and antioxidant activity. *Journal of the Science of Food and Agriculture*.

[69] Huang M, Lin S, Chen C, Pan C, Lee C, Liu H (2013). Oxidative stress status in recently abstinent methamphetamine abusers. *Psychiatry and Clinical Neurosciences*.

[70] Melo P, Zanon-Moreno V, Alves CJ (2010). Oxidative stress response in the adult rat retina and plasma after repeated administration of methamphetamine. *Neurochemistry International*.

[71] Ajjimaporn A, Shavali S, Ebadi M, Govitrapong P (2008). Zinc rescues dopaminergic SK-N-SH cell lines from methamphetamine-induced toxicity. *Brain Research Bulletin*.

[72] Solhi H, Malekirad A, Kazemifar AM, Sharifi F (2014). Oxidative stress and lipid peroxidation in prolonged users of methamphetamine. *Drug Metabolism Letters*.

[73] Teraoka M, Nakaso K, Kusumoto C (2012). Cytoprotective effect of chlorogenic acid against *α*-synuclein-related toxicity in catecholaminergic PC12 cells. *Journal of Clinical Biochemistry and Nutrition*.

